# Breast MRI radiomics: comparison of computer- and human-extracted imaging phenotypes

**DOI:** 10.1186/s41747-017-0025-2

**Published:** 2017-11-21

**Authors:** Elizabeth J. Sutton, Erich P. Huang, Karen Drukker, Elizabeth S. Burnside, Hui Li, Jose M. Net, Arvind Rao, Gary J. Whitman, Margarita Zuley, Marie Ganott, Ermelinda Bonaccio, Maryellen L. Giger, Elizabeth A. Morris, on behalf of the TCGA group

**Affiliations:** 1 Department of Radiology, Memorial Sloan Kettering Cancer Center, 1275 York Ave, New York, NY 10065 USA; 2 Division of Cancer Treatment and Diagnosis, National Cancer Institute, National Institutes of Health, 9609 Medical Center Drive, Rockville, MD 20892 USA; 3 Department of Radiology, University of Chicago, 5841 South Maryland Avenue, MC 2026, Chicago, IL 60637 USA; 4 Department of Radiology, University of Wisconsin School of Medicine and Public Health, 600 Highland Avenue, Madison, WI 53792 USA; 5 Miller School of Medicine, University of Miami, Miami, FL 33136 USA; 6 Department of Bioinformatics and Computational Biology, The University of Texas MD Anderson Cancer Center, Houston, TX 77498 USA; 7 Department of Diagnostic Imaging, The University of Texas MD Anderson Cancer, Center, Houston, TX 77030 USA; 8 Department of Radiology, University of Pittsburgh, Pittsburgh, PA 15213 USA; 9 Department of Radiology, Roswell Park Cancer Institute, Buffalo, NY 14263 USA; 10 300 East 66th Street, New York, NY 10065 USA

**Keywords:** Magnetic resonance imaging, Breast cancer, Inter-observer variability, Machine learning, Radiomics

## Abstract

**Background:**

In this study, we sought to investigate if computer-extracted magnetic resonance imaging (MRI) phenotypes of breast cancer could replicate human-extracted size and Breast Imaging-Reporting and Data System (BI-RADS) imaging phenotypes using MRI data from The Cancer Genome Atlas (TCGA) project of the National Cancer Institute.

**Methods:**

Our retrospective interpretation study involved analysis of Health Insurance Portability and Accountability Act-compliant breast MRI data from The Cancer Imaging Archive, an open-source database from the TCGA project. This study was exempt from institutional review board approval at Memorial Sloan Kettering Cancer Center and the need for informed consent was waived. Ninety-one pre-operative breast MRIs with verified invasive breast cancers were analysed. Three fellowship-trained breast radiologists evaluated the index cancer in each case according to size and the BI-RADS lexicon for shape, margin, and enhancement (human-extracted image phenotypes [HEIP]). Human inter-observer agreement was analysed by the intra-class correlation coefficient (ICC) for size and Krippendorff’s α for other measurements. Quantitative MRI radiomics of computerised three-dimensional segmentations of each cancer generated computer-extracted image phenotypes (CEIP). Spearman’s rank correlation coefficients were used to compare HEIP and CEIP.

**Results:**

Inter-observer agreement for HEIP varied, with the highest agreement seen for size (ICC 0.679) and shape (ICC 0.527). The computer-extracted maximum linear size replicated the human measurement with *p* < 10^−12^. CEIP of shape, specifically sphericity and irregularity, replicated HEIP with both *p* values < 0.001. CEIP did not demonstrate agreement with HEIP of tumour margin or internal enhancement.

**Conclusions:**

Quantitative radiomics of breast cancer may replicate human-extracted tumour size and BI-RADS imaging phenotypes, thus enabling precision medicine.

## Key points

- Computer-extracted breast cancer maximum linear size replicated the human measurement.
- Computer-extracted breast cancer shape replicated the human-extracted imaging phenotypes.
- Using both computer- and human-extracted imaging phenotypes may be clinically valuable.

## Background

Breast magnetic resonance imaging (MRI) is currently the most sensitive imaging modality to detect breast cancer [1]. Radiologists are trained to visually identify pertinent imaging characteristics for cancer detection while taking into account clinical information and context. To standardise reporting and minimise inter-observer variability, the Breast Imaging-Reporting and Data System (BI-RADS), a quality assurance tool, was designed and disseminated into clinical practice [2]. Nevertheless, substantial inter- and intra-observer agreement variability continue to exist among radiologists, though the degree of both is unclear because of a paucity of literature [3]. Despite this recognised variability, the radiologist is the imaging reference standard for interpretation of diagnostic imaging studies, including breast MRI.

The goal of research focusing on integrating computer-aided diagnosis (CAD) and human MRI interpretation is to improve breast cancer detection, moving beyond determining if a lesion is benign or malignant [4–7], and additionally to use radiomics in assessing cancer subtypes. Prototype CAD systems have been investigated clinically and shown to significantly improve the average diagnostic performance of radiologists [8]. Now algorithms are tasked with improving specificity, decreasing observer variability, and identifying biomarkers of breast cancer genotype and/or outcome. Breast cancer radiomics, namely computer-assisted phenotype extraction, can quantify information from voxel-based MRI, providing reproducible information that may be imperceptible to the human eye. Quantitative radiomics can analyse texture features, providing further insight into inter- and intra-tumour heterogeneity and assess enhancement kinetics [3, 7, 9–11]. Similar to human assessments, there may be computer software and/or algorithm variability.

Diagnostic breast MRI is in the midst of a paradigm shift whereby the correlative and complementary roles of human-extracted imaging phenotypes (HEIP) and computer-extracted imaging phenotypes (CEIP) are being assessed for the diagnosis, treatment and management of breast cancer. There may soon be a clinical role for using both HEIP and CEIP wherein the strengths of each are leveraged to generate combined biomarkers that accurately predict important outcomes. One of the initial steps for CEIP to be clinically accepted is to compare them with the clinical gold standard (i.e., human radiologists) and to demonstrate that CEIP can reasonably replicate HEIP. The purpose of this study was to investigate if CEIP MRI phenotypes of breast cancer could replicate HEIP (i.e., human-extracted size and BI-RADS phenotypes) using MRIs from The Cancer Genome Atlas (TCGA) project of the National Cancer Institute.

## Methods

### Institutional review board approval

In this retrospective study, all patient data were Health Insurance Portability and Accountability Act-compliant and acquired under institutional review board approval with a waiver of the need for informed consent.

### Patient population

We retrieved data from an open-source de-identified database, The Cancer Imaging Archive (TCIA) [12], which is the imaging counterpart of TCGA. TCGA, in brief, is a coordinated effort led by the National Cancer Institute to accelerate the molecular and genomic understanding of cancer [13]. The TCGA program performs genomic sequencing and characterisation of tissue from cancers diagnosed and treated at cancer centres around the United States.

Breast cancer (both invasive ductal and lobular) was one of the cancers selected for study, and by December 16, 2014, 1100 breast cancer cases had been collected by TCGA and 1098 also had available genomic and clinical data (available at: https://gdc.cancer.gov/; accessed February 17, 2016). To complement the genomic and clinical data, the National Cancer Institute enriched the TCGA open-source data portal by collecting MRI studies for storage and analysis in the TCIA. However, in 2014, only 108 breast cancers had TCIA MRI (pre-operative examinations with pathologically verified cancer) to correlate with available TCGA data. Clinical, pathologic and genomic data were extracted using the TCGA assembler, an open-source, publicly available, free tool [14].

Because our patients were drawn from the TCGA, an open-source database, some patients in our cohort were included in several previously published studies [9–11, 15, 16]. However, this paper has no scientific overlap with the other published studies. We report new findings from our study done to investigate if CEIP of breast cancer could replicate HEIP using MRI from TCGA. Thus, whereas we assessed the correlation between CEIP and HEIP, the other studies assessed the correlation between CEIP and clinical features.

### MRI acquisition

All breast MRI data were downloaded from the Breast Cancer Risk Assessment collection within TCIA (http://www.cancerimagingarchive.net). The data had previously been generated under MRI studies originally performed between 1999 and 2004 at four institutions: the Mayo Clinic, Memorial Sloan Kettering Cancer Center, Roswell Park Cancer Institute and the University of Pittsburgh Medical Center. To avoid differences in image quality caused by different equipment vendors, only MR images obtained with 1.5-T systems were included in our study, which limited the number of patients considered for inclusion in our analysis to 93. Of these 93 patients, two were excluded because of missing genomic data (*n* = 1) and missing images (*n* = 1).

Therefore, in total, 91 female patients with pre-operative breast MRI studies underwent analysis in our study. All images were acquired with a 1.5-T system (Signa or Signa HDX; General Electric Medical Systems, Waukesha, WI, USA). In all patients, a dedicated surface breast coil was used. T1-weighted fat-suppressed images were acquired before and after intravenous administration of a gadolinium-based contrast agent (gadodiamide, Omniscan®; Nycomed-Amersham, Princeton, NJ, USA), with three to five post-contrast images because the protocol was institution-dependent. In-plane spatial resolution ranged from 0.53 to 0.86 mm, and slice spacing ranged from 2 to 3 mm. Only pre- and post-contrast T1-weighted fat-suppressed images were included in our study. Further information, including full clinical breast MRI protocols, can be accessed from the open-source TCIA.

### HEIP

To generate HEIP, a pool of eleven board-certified breast-imaging radiologists, with experience ranging from 4 to 29 years (ESB, 14 years; GJW, 25 years; EJS, 4 years; JMN, 5 years; MG, 29 years; and EAM, 25 years; the other five radiologists were non-authors), participated in the manual assessment of MRI data. The image location of the index breast cancer and maximal tumour size were identified on the first post-contrast image and annotated by each radiologist using an open-source and open-access software platform [17]. For each patient, three radiologists from this pool were randomly assigned to review the imaging data. Visual assessments of tumour characteristics of the cancer were made according to BI-RADS 5 descriptors (lesion-shaped, internal enhancement and margin), yielding HEIP. In patients with multifocal or multicentric disease, the largest mass was used as the index lesion.

Four HEIP characteristics were assessed: lesion size (largest size as per Response Evaluation Criteria in Solid Tumours 1.1 recommendation [18]), lesion shape (whether the lesion was irregularly shaped or round/oval), internal enhancement (whether the enhancement was heterogeneous or homogeneous) and margin (whether the margin was circumscribed, irregular or spiculated). The radiologists performed their reviews independently and were blinded to all health information. Enhancement kinetics/curves were not generated, owing to the variable temporal resolution across sites and over time, which would have significantly impacted the accuracy of the results. HEIP for each feature from the three radiologists was summarised into a single representative consensus value for each patient; these summary values served as the case-based HEIP in the subsequent statistical analysis. The decision rule for consensus was simple majority. There were no cases where all readers disagreed.

### CEIP (quantitative radiomics)

Given the approximate tumour centre location, each index breast tumour was automatically segmented in the three-dimensional (3D) space. The quantitative radiomics workstation used for this study was able to yield 38 CEIP from dynamic contrast-enhanced MRI scans to characterise tumour size, shape, margin, enhancement texture, kinetics, and variance kinetics [6, 7, 19–21]. However, because kinetic characteristics were not assessed by the radiologists (i.e., as HEIP) for this study, only 24 phenotypes (from four categories) were used to compare the HEIP and CEIP. The 14 kinetics-related CEIP were excluded. All measurements were extracted from the first post-contrast MR images.

**Fig. 1:**
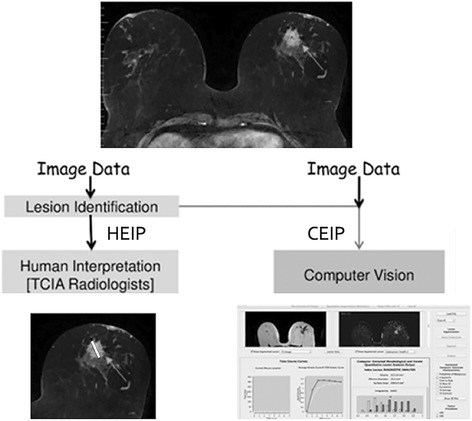
Schematic of breast magnetic resonance imaging human-extracted image phenotypes (HEIP) and computer-extracted imaging phenotypes (CEIP). *TCIA* The Cancer Imaging Archive

The 24 CEIP were calculated on the basis of automatically derived 3D tumour segmentations [19]. They were further separated into four phenotypic categories: (a) size-measuring tumour dimensions (4 CEIP); (b) shape, quantifying the 3D tumour geometry (3 CEIP); (c) morphology, combining tumour shape and margin characteristics such as margin sharpness (3 CEIP); and (d) enhancement texture, describing the texture of the contrast uptake (heterogeneity of the uptake) in the tumour on the first post-contrast MRI image (14 CEIP) (Fig. 1). Owing to our small sample size, we report only the CEIP extracted for each HEIP BI-RADS descriptor but do not provide the coefficients.

### Statistical methods

#### Inter-observer agreement in HEIP

Inter-observer agreement for human-extracted lesion size was based on inferences on the (−log 1.20, log 1.20) coverage probability of the natural logarithm of the size. This is the probability that the absolute difference between the natural logarithm of the size measurements from any two radiologists differs by less than log 1.20, or equivalently, that the size measurements from any two radiologists are within 20% of each other. Inter-observer agreement for human-extracted tumour shape, margin and heterogeneity was based on inferences on Krippendorff’s α [22]. The 95% CIs for Krippendorff’s α and the coverage probability were constructed using the nonparametric bootstrap [23].

#### Associations between HEIP and CEIP

Associations for lesion size were assessed through inferences on the Kendall τ rank correlation coefficient [24]; *p* values were obtained through permutation tests. Associations for both shape and internal enhancement were assessed through the Mann-Whitney *U* test [25]. Associations for margin were assessed through inferences on the Kendall τ rank correlation coefficient; *p* values were obtained through permutation tests [26]. The Benjamini-Hochberg procedure was used to correct for multiple hypothesis testing. All tests of associations between HEIPs and individual CEIPs were performed at the α = 0.05 level [27].

#### Replicating HEIP using CEIP

We analysed the feasibility of replicating each of the four HEIP using the corresponding CEIP. These were treated as either prediction or classification problems that we evaluated in terms of accuracy. The replication of HEIP of tumour size given the CEIP of tumour size was treated as a prediction problem. A predictor for the HEIP of tumour size based on the size-related CEIP was constructed using multivariate linear regression subject to elastic net constraints [28]. The elastic net constraints set some coefficients in the model exactly to zero because of the model’s geometry, thus performing variable selection, and also simultaneously stabilises the coefficient estimates in the presence of substantial correlation between the CEIP. Ten-fold cross-validation [29] was used to select the values of the tuning parameters controlling the severity of the elastic net constraints.

The replications of tumour shape, internal enhancement, and margin were treated as classification problems. Classifiers for tumour shape and internal enhancement were constructed using multiple logistic regression subject to elastic net constraints. Classifiers for tumour margin were constructed using ordinal logistic regression, also subject to elastic net constraints. For these classifiers, tuning parameters for the elastic net constraints were also selected using ten-fold cross-validation.

These predictors and classifiers were then assessed in terms of their prediction and classification accuracy through nested ten-fold by ten-fold cross-validation [30]. For tumour size, performance was evaluated in terms of the mean squared deviation [31]. For tumour shape and internal enhancement, performance was evaluated in terms of AUC in ROC analysis. For tumour margin, performance was evaluated in terms of the Kendall τ rank correlation coefficient between the classifier score and the actual value of the HEIP of tumour margin [24].

For each of these four tumour characteristics (size, shape, internal enhancement, and margin), the *p* values of predictive signal contained within CEIP were computed through permutation tests. The Benjamini-Hochberg procedure was applied to these *p* values to correct for multiple hypothesis testing [27].

## Results

### Patients

Ninety-one patients with invasive breast cancer who underwent pre-operative breast MRI were included in the study sample. Their median age was 53.6 years (range 29–82) at first cancer diagnosis (Table 1). Breast cancers were predominantly oestrogen receptor- and progesterone receptor-positive. Human epidermal grow factor receptor 2 status was known only in 63 (69%) of 91 patients, of whom 49 (78%) of 63 were negative and 14 (22%) of 63 were positive. Additional information regarding the tumours can be found on the open-source website (available at: https://gdc.cancer.gov/; accessed February 17, 2016).

**Table 1:** Patient and invasive breast cancer characteristics and axillary lymph node status

Characteristics	Total (*N* = 91), *n* (%)
Mean age, years (range)	53.6 (29–82)
Tumour diameter, cm (SD)	2.41 (0.78–5.93)
Invasive breast	
Ductal carcinoma	79 (86.8%)
Lobular carcinoma	10 (11.0%)
Other	2 (2.2%)
Oestrogen receptor	
Positive	76 (83.5%)
Negative	15 (16.5%)
Progesterone receptor	
Positive	72 (79.1%)
Negative	19 (20.9%)
HER2 receptor	
Positive	14 (15.4%)
Negative	49 (53.8%)
Unknown	28 (30.8%)
Lymph node status	
Positive	44 (48.4%)
Negative	46 (50.5%)
Unknown	1 (1.1%)
Stage	
I	22 (24.2%)
II	58 (63.7%)
III	11 (12.1%)

*HER2* Human epidermal grow factor receptor 2

Numbers in parentheses represent percent for categorical variables unless otherwise indicated

### Inter-observer agreement in HEIP

There was variability in the inter-observer agreement of HEIP of tumour size, shape, margin and internal enhancement (Table 2). Tumour size, the only quantitative measurement, showed the strongest inter-observer agreement, with a coverage probability of π = 0.679 (95% CI 0.561–0.736). Tumour shape and margin showed moderate inter-observer agreement, with α = 0.527 (95% CI 0.380–0.654) and α = 0.561 (95% CI 0.426–0.674), respectively. Tumour internal enhancement showed the poorest inter-observer agreement, with α = 0.292 (95% CI 0.147–0.433).

**Table 2:** Human-extracted imaging phenotypes and inter-observer agreement

HEIP	Inter-observer agreement (95% CI)
Lesion size	π = 0.679 (0.561–0.736)
Shape	α = 0.527 (0.380–0.654)
Internal enhancement	α = 0.292 (0.147–0.433)
Margin	α = 0.561 (0.426–0.674)

*HEIP* Human-extracted image phenotype

### Association between HEIP and CEIP

Strong evidence of associations between the HEIP and corresponding CEIP were observed in some cases, indicating that the CEIP may have the ability to replicate the HEIP (Table 3).

**Table 3:** Association between human-extracted image phenotype and computer-extracted image phenotype

Human-extracted assessment	Computer-extracted feature	*p* Value
Lesion size	Effective diameter	<10^−12^
	Surface area-to-volume ratio	<10^−12^
	Maximum linear size	<10^−12^
	Lesion volume	<10^−12^
Shape	Sphericity	5.65 × 10^−5^
	Irregularity	0.00398
	Surface area-to-volume ratio	0.363
Internal enhancement	Contrast	0.378
	Correlation	0.409
	Difference in entropy	0.353
	Difference variance	0.191
	Energy	0.186
	Entropy	0.194
	Inverse difference moment	0.466
	IMC1	0.328
	IMC2	0.340
	Maximum correlation coefficient	0.336
	Sum average	0.151
	Sum entropy	0.336
	Sum variance	0.702
	Sum of squares	0.297
Degree of margin spiculation	Mean margin sharpness	0.292
	Variance margin sharpness	0.227
	Variance radial gradient histogram	0.055

*IMC* Information measure of correlation

**Fig. 2:**
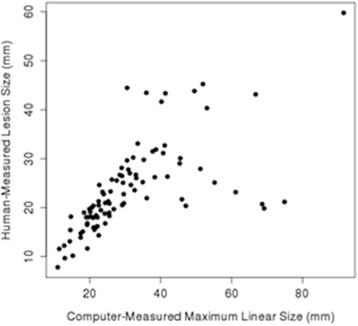
Comparison between human- and computer-measured maximum linear size

**Fig. 3:**
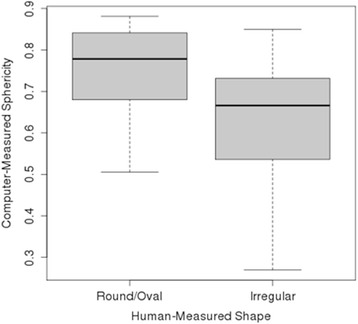
Comparison of computer- and human-extracted shapes

**Fig. 4:**
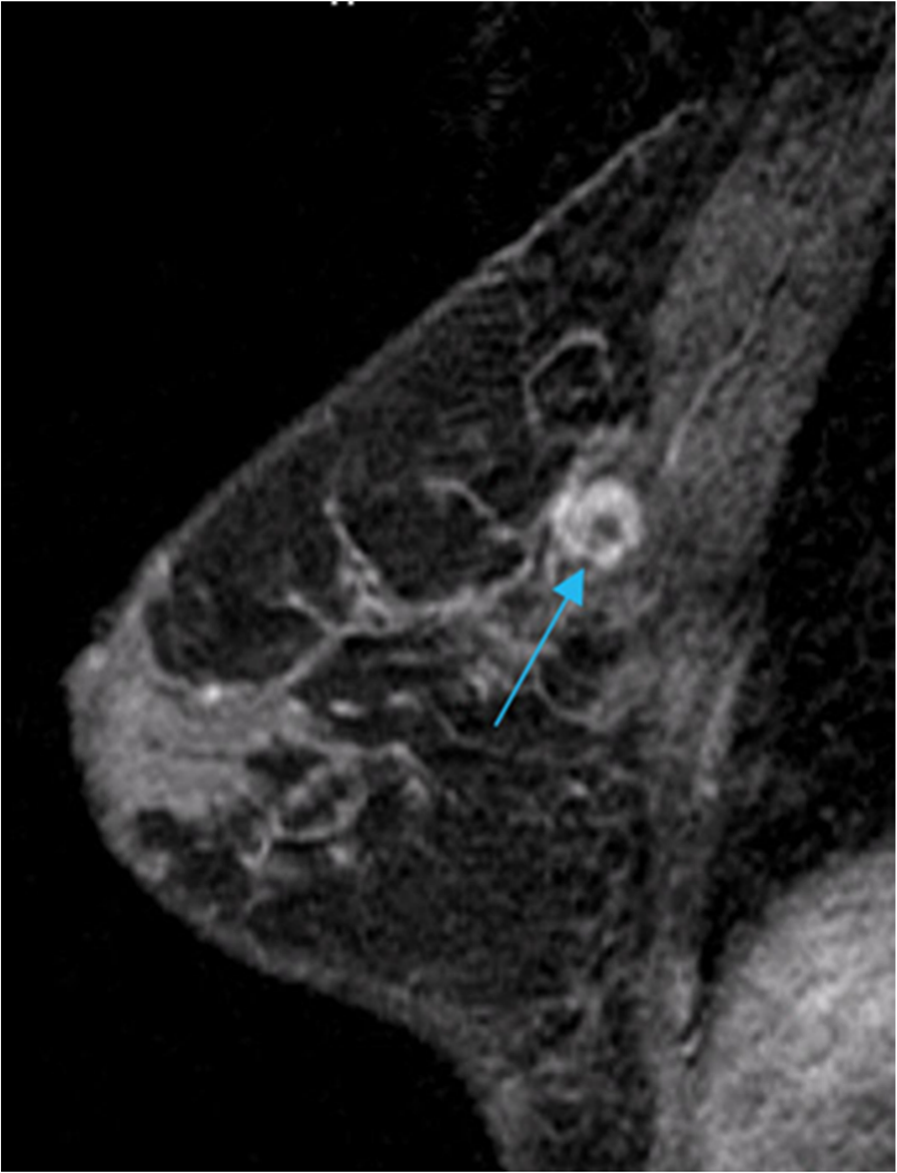
Sagittal fat-suppressed T1-weighted first post-contrast image of a breast cancer (arrow) where computer-extracted image phenotype (CEIP) sphericity is high and the radiologist assessed it as round/oval for shape. CEIP and human-extracted image phenotype are concordant

**Fig. 5:**
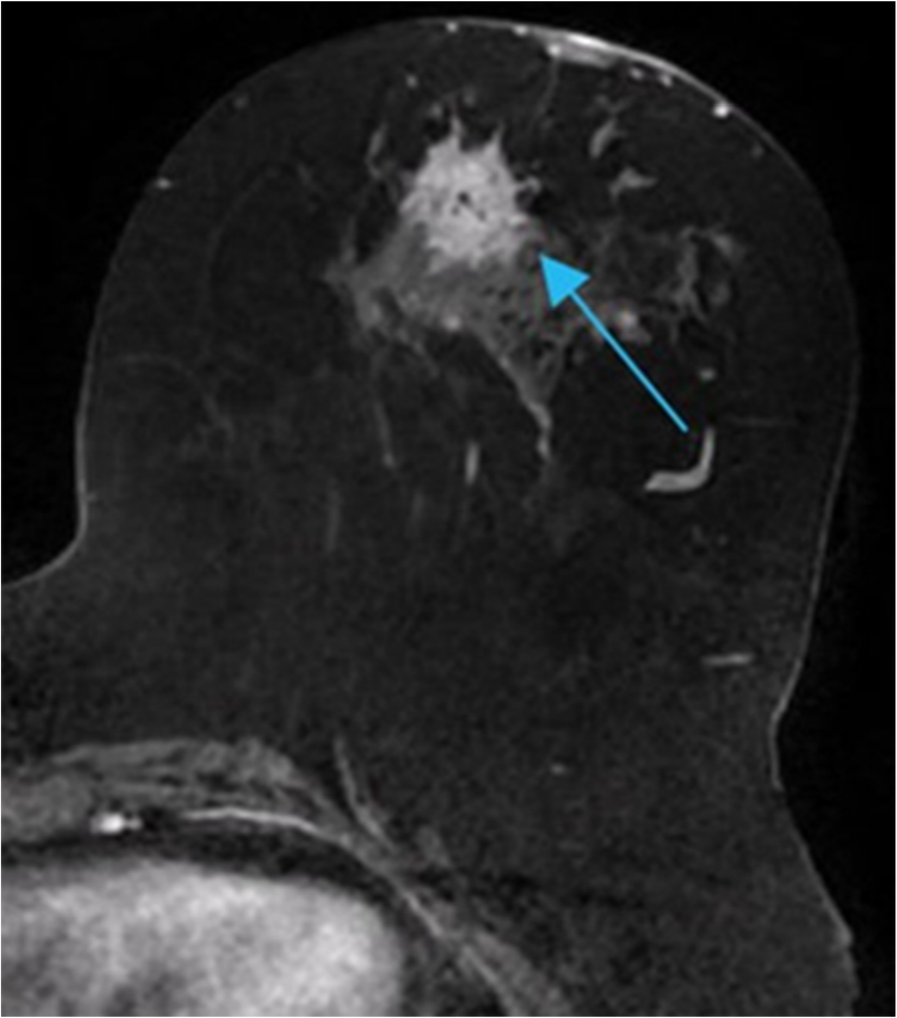
Axial fat-suppressed T1-weighted first post-contrast image of a breast cancer (arrow) where computer-extracted image phenotype (CEIP) sphericity is high and the radiologist assessed it as irregular for shape. CEIP and human-extracted image phenotype are discordant

The *p* values of the associations between HEIP of tumour size and each of the size-related CEIPs were all < 10^−12^. Figure 2 shows a scatterplot of the human-extracted tumour size versus each of the four tumour size-related CEIP. The *p* values of the associations between the HEIP and corresponding CEIP of tumour shape, specifically sphericity, and irregularity, were 5.65 × 10^−5^ and 3.98 × 10^−3^, respectively. Figure 3 shows box plots of the CEIP of tumour sphericity and irregularity versus HEIP of tumour shape. Figures 4 and 5 demonstrate representative cases where CEIP and HEIP are concordant and discordant, respectively. These associations also remained significant even after adjustment for multiple comparisons using the Benjamini-Hochberg procedure [27].

Meanwhile, no evidence of association between the HEIP and the corresponding CEIP of tumour internal enhancement or tumour margin was observed.

### Feasibility of replicating HEIP using combinations of HEIP

A summary of the results of the analysis of the ability of CEIP to replicate HEIP is given in Table 4.

**Table 4:** Abilities of computer-extracted image phenotypes to replicate corresponding human-extracted phenotypes

BI-RADS feature	Performance metric estimate with *p* value	CEIPs involved in chosen predictor/classifier
Lesion size	MSD 56.558 (*p* < 0.001)	Effective diameter Surface area Maximum linear size
Lesion shape	AUC 0.720 (*p* = 0.004)	Sphericity
Internal enhancement	AUC 0.551 (*p* = 0.263)	Energy Entropy IMC1 Maximum correlation coefficient Sum of average Sum of entropy Sum of squares
Margin	τ = 0.143 (*p* = 0.183)	Variance radial gradient histogram

*IMC* Information measure of correlation, *MSD* Mean squared deviation

HEIP of tumour size could be replicated using a weighted sum of CEIP of effective diameter, surface area and maximum linear size (mean squared deviation 56.56, *p* < 0.001). HEIP of tumour shape could also be replicated using CEIP of tumour sphericity (AUC 0.720, *p* = 0.004). Both the abilities of tumour size-related CEIP to replicate HEIP of tumour size and CEIP of tumour sphericity to replicate HEIP of tumour shape remained statistically significant even after correction for multiple testing.

The HEIP of tumour internal enhancement could not be replicated with a reasonable degree of accuracy using CEIP of tumour enhancement; the AUC associated with the best model as identified through ten-fold cross-validation, which involved energy, entropy, information measure of correlation, maximum correlation coefficient, sum of average, sum of entropy, and sum of squares, was 0.551 (*p* = 0.263).

The HEIP of tumour margin could not be replicated with a reasonable degree of accuracy using CEIP of tumour margin. The value of τ associated with the best classifier as identified through ten-fold cross-validation, which involved variance of the radial gradient histogram only, was 0.143 (*p* = 0.183). Because of the limited sample size of 91 and the preliminary nature of this analysis, only the CEIP involved in the predictor or classifier of the HEIP BI-RADS descriptors is reported.

## Discussion

Our results demonstrate that breast MRI CEIP can replicate some HEIP of breast cancer. In our study, we evaluated four HEIP characteristics: lesion size, lesion shape, internal enhancement, and margin. Specifically, CEIP in our study was able to replicate radiologists’ assessment of tumour size and shape. There was a correlation between the CEIP and radiologists’ measurement of tumour margins; however, it was not statistically significant.

For radiologists in the era of precision medicine, the quantification and precision of radiology using computerised MRI analysis (i.e., radiomics) are a rapidly evolving field of interest, paralleling the pace of technologic innovation outside healthcare. Computer machine-learning algorithms continue to be developed to facilitate tumour diagnosis (detection and localisation), segmentation (automated and semi-automated), and feature extraction on MRI. Radiomics of breast cancer on MRI, which involves the extraction of quantitative imaging features (also known as *CEIP*), have the potential to provide further insight into tumour imaging phenotypes that are imperceptible to the human eye [32, 33]. In contrast to the qualitative nature of HEIP, these quantitative characteristics of CEIP may improve the reproducibility, accuracy and predictive power of existing subjective BI-RADS terms. Furthermore, computers do not suffer from fatigue, distraction or hunger, which are known to affect the clinical performance of humans [34, 35]. In addition, to the advantages mentioned above, CEIP may be able to identify new image features that could become biomarkers of tumour biology, predictors of treatment response or surrogates for genetic testing [32, 33].

Some correlations between CEIP and breast cancer subtype and genotype have been reported in the literature. Bhooshan et al [36] developed CEIP as MRI-based prognostic markers, distinguishing between ductal carcinoma in situ and invasive breast cancer and between breast cancer with and without positive lymph nodes. They [37] also demonstrated CEIP in the classification of breast cancer tumour grades. Mazurowski et al [38] found that the Luminal B molecular subtype is associated with enhancement dynamics on MRI. Sutton et al [39] developed a predictive model using features extracted from MRI that could distinguish between invasive ductal carcinoma molecular subtypes. Agner et al [40] reported that CEIP could identify triple-negative breast cancers and could differentiate them from other molecular subtypes. Sutton et al [41] developed a model using CEIP that could predict the likelihood of recurrence and magnitude of chemotherapeutic benefit. The TCGA Breast Phenotype Group has related CEIP to clinical features including pathologic stage [11]; molecular classification of breast cancers (such as luminal A) [15]; risk of recurrence with research versions of Oncotype DX, MammaPrint and PAM50 multi-gene assays [9]; clinical phenotype [10]; and genetics of various pathways [16].

The results of this study suggest a potential role for CEIP tumour features; however, further research must demonstrate how CEIP can assist, complement, or benefit the human/radiologist. In our study, inter-observer variability in radiologists’ use of tumour BI-RADS features was significant even among experts in breast imaging. The highest correlation was seen with size and shape. These features were also those that the CEIP combinations could successfully replicate. The difficulty in our study for CEIP in replicating HEIP of tumour internal enhancement or margin may be a result of low radiologist inter-observer agreement; it is hard to predict features that radiologists do not agree on. The reason for low inter-observer agreement is unclear, and there is a paucity of literature exploring this [3]. All readers in thus study were fellowship-trained breast-imaging radiologists. Future research could be focused on reproducible quantitative CEIP tumour features and their possible role as the imaging reference standard for BI-RADS HEIP with high inter-reader variability, such as tumour internal enhancement and margin in our study, also possibly concealing clinically important information. Before this can happen, however, the research community must decide on a standardised computer algorithm for tumour segmentation and quantitative image analysis that work across different image protocols, image quality and magnet strength. A CEIP lexicon of breast cancer image features analogous to BI-RADS is needed because features that can be extracted by computers are vast and somewhat abstract to the clinical radiologist who is used to the BI-RADS lexicon. Finally, there may be variability in CEIP tumour assessments analogous to the inter- and intra-observer variability seen with radiologists.

The present study had several limitations. First, this was a retrospective analysis of 91 patients who presented over 6 years across 4 different institutions. Image acquisition took place between 1999 and 2004, and technology has since improved significantly. Most patients had oestrogen receptor- and progesterone receptor-positive breast cancers; therefore, our evaluation was not representative of all immunohistochemical subtypes. In addition, all index lesions were masses, so it remains to be determined if the same results would be seen with cancers presenting as only non-mass enhancement. Second, the standard MRI acquisition parameters varied across institutions and possibly over time. Third, it is uncertain if our results can be extrapolated for different imaging equipment and protocols. Finally, this is a small dataset, and our classifier cannot be assumed to work on a larger dataset.

In conclusion, this study we found that, for some phenotypes, CEIP were able to successfully replicate breast MRI HEIP. Thus we propose that there may be a role in the future for using both whereby the strengths of each are leveraged to optimise diagnostic reporting, decrease inter-observer variability, and perhaps even improve our understanding of breast cancer. As computer algorithms continue to be developed, we foresee that radiology reports in the future will include quantitative metrics besides size, the result of CEIP from validated computer algorithms. This information will be compounded by the continuing improvements in MRI sequences, specifically the ability to perform simultaneously high temporal and spatial resolution imaging. Our study demonstrates the feasibility of a future method where radiomics are part of everyday radiology reporting. Future research will be needed on larger datasets to validate this observation. In addition, the observed trend that the correlation between CEIP and HEIP corresponded to the degree of inter-observer agreement requires further investigation. Large-scale datasets are needed to provide further evidence for the use of CEIP in clinical practice.

## Abbreviations

BI-RADS: Breast Imaging-Reporting and Data System
CAD: Computer-aided diagnosis
CEIP: Computer-extracted image phenotype
3D: Three-dimensional
HEIP: Human-extracted image phenotype
ICC: Intra-class correlation coefficient
MRI: Magnetic resonance imaging
MSD: Mean squared deviation
TCGA: The Cancer Genome Atlas
TCIA: The Cancer Imaging Archive
